# Circulating microRNAs in plasma of patients with gastric cancers

**DOI:** 10.1038/sj.bjc.6605608

**Published:** 2010-03-16

**Authors:** M Tsujiura, D Ichikawa, S Komatsu, A Shiozaki, H Takeshita, T Kosuga, H Konishi, R Morimura, K Deguchi, H Fujiwara, K Okamoto, E Otsuji

**Affiliations:** 1 Division of Digestive Surgery, Department of Surgery, Kyoto Prefectural University of Medicine, 465 Kajii-cho, Kawaramachihirokoji, Kamigyo-ku, Kyoto 602–8566, Japan

**Keywords:** gastric cancer, microRNA, plasma, biomarker

## Abstract

**Background:**

We examined plasma microRNA (miRNA) concentrations from patients with gastric cancers (GCs) to assess their clinical application for diagnosing and monitoring diseases.

**Methods:**

We initially investigated the appropriateness of plasma miRNA assay, and then compared plasma miRNA results with the expressions in cancer tissues from eight GC patients, and also compared plasma miRNAs between pre- and post-operative paired samples from 10 GC patients. Then, plasma miRNAs (*miR-17-5p*, *miR-21*, *miR-106a*, *miR-106b* and *let-7a*) were analysed in 69 GC patients and 30 healthy volunteers in total.

**Results:**

The initial analysis showed that miRNAs were stable and detectable in all plasma samples, and the plasma miRNA levels reflected the tumour miRNAs in most cases. The levels of these miRNAs were significantly reduced in post-operative samples. In large-scale analysis, the plasma concentrations of miRNAs (*miR-17-5p*, *miR-21*, *miR-106a*, *miR-106b*) were significantly higher in GC patients than controls (*P*=0.05, 0.006, 0.008 and <0.001 respectively), whereas *let-7a* was lower in GC patients (*P*=0.002). The values of the area under the receiver-operating characteristic curve were 0.721 for the *miR-106b* assay and 0.879 for the *miR-106a*/*let-7a* ratio assay.

**Conclusion:**

Detection of circulating miRNAs might provide new complementary tumour markers for GC.

Gastric cancer (GC) is the second leading cause of cancer-related death in the world (Parkin *et al*, 2005). Recent advances in diagnostic techniques and peri-operative management have increased the early detection of GC and decreased the mortality rate. However, patients with advanced disease still frequently develop recurrent disease after extended radical resections, and consequently show extremely poor survival rates (Martin *et al*, 2002). Thus, the primary tumours must be detected at an early stage, and recurrent disease must be diagnosed when it is still minimal or clinically occult, to improve the cure rates for patients with GCs.

For GC, serum tumour markers, such as carcinoembryonic antigen and carbohydrate antigen 19-9, have been used as convenient diagnostic assays (Nakane *et al*, 1994; Marrelli *et al*, 2001). These conventional serum markers, however, lack sufficient sensitivity and specificity to facilitate early detection of cancer. In addition, there are no other less invasive diagnostic tests for GC such as faecal occult blood tests for colon cancer.

Recently, several studies have shown that microRNAs (miRNAs), which are involved in tumourigenesis and the development of various cancers, are stably detectable in plasma/serum (Calin and Croce, 2006; Chen *et al*, 2008; Filipowicz *et al*, 2008). Mitchell *et al* (2008) clearly showed that circulating miRNAs originate from cancer tissues, and are protected from endogenous RNase activity by unknown mechanisms. They also showed the potential of using plasma/serum miRNAs as a non-invasive blood-based biomarker for the detection of prostate cancer. These findings have opened up a new and interesting field in the screening and monitoring of cancer patients.

In this study, we investigated the amounts of circulating miRNAs in plasma samples from both pre-operative GC patients and controls, and compared the relationships between the results and clinical findings to assess the diagnostic value of these biomarkers in patients with GCs.

## Materials and methods

### Patients and samples

Pre-operative plasma samples were collected from 34 patients with GCs, who underwent gastrectomy at Kyoto Prefectural University of Medicine, as well as from 15 healthy volunteers for test-scale analysis. To evaluate the appropriateness of this plasma miRNA assay, we initially investigated the level of three miRNAs, such as *miR-21*, *miR-106b* and *let-7a*, in plasma samples by real-time RT-PCR assay using the synthetic miRNAs, mirVana miRNA Reference Panel (Ambion, Austin, TX, USA). Thereafter, we performed two experiments to examine whether the plasma miRNAs could reflect tumour dynamics. First, formalin-fixed paraffin-embedded tumour samples were collected from eight patients with higher levels of plasma *miR-106b* and lower levels of plasma *let-7a* than healthy volunteers, and compared the miRNA expressions in primary lesions with those in plasma samples. Second, paired plasma samples before and 1 month after gastrectomy were collected from 10 of the test-scale patients, and findings in pre- and post-operative plasma samples were compared. The initial experiments showed that plasma miRNA assays were feasible and could reflect tumour dynamics, and then we performed a large-scale validation in the next step.

Pre-operative plasma samples were collected from another 35 patients with GCs for further large-scale analyses, as well as from another 15 healthy volunteers. Thus, between October 2008 and July 2009, 69 patients with GC and 30 healthy volunteers were enrolled in this study. We added another two miRNAs, *miR-17-5p* and *miR-106a*, as candidates for biomarkers for large-scale analyses. A peripheral blood sample was collected from patients and volunteers after obtaining informed consent and agreement. Immediately after collection, the blood samples were subjected to isolation of cell-free nucleic acids using a three-spin protocol (1500 r.p.m. for 30 min, 3000 r.p.m. for 5 min, 4500 r.p.m. for 5 min) to prevent contamination by cellular nucleic acids. Plasma samples were then stored at −80°C until further processing. None of the patients had received chemotherapy or radiotherapy before blood sampling. The resected GC specimens were fixed in buffered formalin and embedded in paraffin for pathological examination by standard methods. Macroscopic and microscopic classifications of tumours were based on the IGCC/TMN staging system (Sobin and Wittekind, 2002).

### RNA extraction

Total RNA was extracted from 400 *μ*l of plasma using mirVana PARIS Kit (Ambion), and finally eluted into 100 *μ*l of pre-heated (95°C) Elution Solution according to the manufacturer's protocol. As for formalin-fixed paraffin-embedded tissues, total RNA was extracted from four slices 15 *μ*m thick (total 60 *μ*m in thickness) using RecoverAll Total Nucleic Acid Isolation Kit (Ambion), and eluted finally into 60 *μ*l of Elution Solution according to the manufacturer's protocol.

### Protocols for the detection of miRNAs

The amounts of miRNAs were quantified in duplicate by qRT-PCR using the human TaqMan MicroRNA Assay Kits (Applied Biosystems, Foster City, CA, USA). The reverse transcription reaction was carried out with TaqMan MicroRNA Reverse Transcription Kit (Applied Biosystems) in 15 *μ*l containing 5 *μ*l of RNA extract, 0.15 *μ*l of 100 mM dNTPs, 1 *μ*l of Multiscribe Reverse Transcriptase (50 U *μ*l^−1^), 1.5 *μ*l of 10 × reverse transcription buffer, 0.19 *μ*l of RNase inhibitor (20 U *μ*l^−1^), 1 *μ*l of gene-specific primer and 4.16 *μ*l of nuclease-free water. For synthesis of cDNA, the reaction mixtures were incubated at 16°C for 30 min, at 42°C for 30 min, at 85°C for 5 min and then held at 4°C. Then, 1.33 *μ*l of cDNA solution was amplified using 10 *μ*l of TaqMan 2 × Universal PCR Master Mix with no AmpErase UNG (Applied Biosystems), 1 *μ*l of gene-specific primers/probe and 7.67 *μ*l of nuclease-free water in a final volume of 20 *μ*l. Quantitative PCR was run on a 7300 Real-Time PCR system (Applied Biosystems) and the reaction mixtures were incubated at 95°C for 10 min, followed by 40 cycles of 95°C for 15 s and 60°C for 1 min. The cycle threshold (*C*_t_) values were calculated with the SDS 1.4 software (Applied Biosystems).

The amounts of plasma miRNAs were calculated on a standard curve constructed with the use of synthetic miRNAs, mirVana miRNA Reference Panel (Ambion). The standard reference miRNAs were amplified for each reaction. However, the expression of miRNAs from tissue samples was normalised using the 2−*ΔΔC*_t_ method relative to RNU6B. The *ΔC*_t_ was calculated by subtracting the *C*_t_ values of RNU6B from the *C*_t_ values of the miRNAs of interest. The *ΔΔC*_t_ was then calculated by subtracting *ΔC*_t_ of the surrounding normal gastric epithelium from *ΔC*_t_ of cancer tissues. Fold change in the gene was calculated by the equation 2-*ΔΔC*_t_ (Livak and Schmittgen, 2001; Pfaffl, 2001).

### Statistical analysis

Mann–Whitney test was used to compare the difference in plasma miRNA concentration and miRNA ratio between the cancer group and the healthy group, and Wilcoxon test was used to compare the paired plasma samples before and 1-month after gastrectomy. *P*-value <0.05 was considered significant. Receiver-operating characteristic (ROC) curves and the area under the ROC curve (AUC) were used to assess the feasibility of using plasma miRNA concentration as diagnostic tools for detecting GC. We used the Youden index for identification of the optimal cut-off point.

## Results

### Evaluation of quantitative RT-PCR for measuring the miRNAs in plasma sample

**Figure 1:**
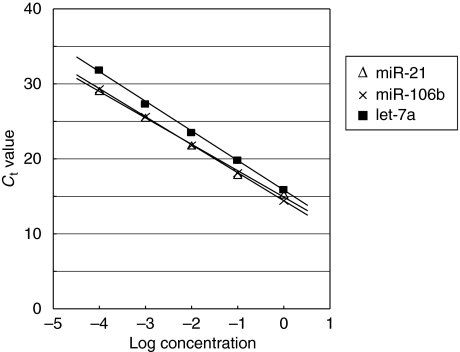
Standard curve of *miR-21*, *miR-106b*, *let-7a* using synthetic miRNAs. Ten-fold serial dilution of synthetic miRNA was used to generate the standard curves. Linearity was confirmed within these concentrations, ranging from 1 to 0.0001 fmol. (*miR-21*: *y*=−3.7544*x*+14.318 (*R*^2^=0.999); *miR-106b*: *y*=−3.9849*x*+15.645 (*R*^2^=0.998); *let-7a*: *y*=−3.4988*x*+14.875 (*R*^2^=0.999))

**Figure 2:**
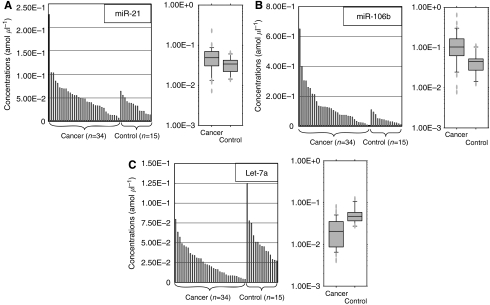
Plasma miRNAs concentration in the initial analysis. Real-time RT-PCR assay, circulating plasma miRNAs (**A**: *miR-21*, **B**: *miR-106b* and **C**: *let-7a*) were detectable and amplified in all samples from 34 gastric cancer patients and 15 healthy volunteers. The concentrations of *miR-106b* and *let-7a* were significantly higher and lower in plasma from gastric cancer patients than in that from healthy controls (*P*=0.002 and <0.001 respectively). However, there was no significant difference in the concentration of *miR-21* between gastric cancers patients and controls although it tended to be higher in gastric cancer patients (*P*=0.088). The upper and lower limits of the boxes and the lines inside the boxes indicate the 75th and 25th percentiles and the median respectively. The upper and lower horizontal bars denote the 90th and 10th percentiles respectively.

To evaluate the appropriateness of this plasma assay, we first conducted amplification by real-time RT-PCR assay of a 10-fold serial dilution of the mirVana miRNA Reference Panel. The linearity of the quantitative RT-PCR was confirmed from the concentrations of 1 to 0.0001 fmol of each synthetic miRNAs, such as *miR-21*, *miR-106b* and *let-7a* (*R*^2^=0.999, 0.998 and 0.999 respectively) between the logarithm of the amount of input miRNAs and the *C*_t_ values (Figure 1). Using this assay, we found circulating miRNAs (*miR-21*, *miR-106b*, and *let-7a*) detectable and amplified in all samples from 34 GC patients and 15 healthy volunteers. The concentrations of *miR-106b* were significantly higher, whereas those of *let-7a* were significantly lower in plasma from GC patients than in that from healthy controls (*P*=0.002 and <0.001 respectively; Figure 2B and C). However, there was no significant difference in the concentration of *miR-21* between GC patients and controls, although it tended to be higher in GC patients (*P*=0.088; Figure 2A).

### Relationship between the miRNAs in plasma and primary gastric cancer tissues

There were eight patients whose *miR-106b* concentrations exceeded the highest level of healthy volunteers, and let-7a concentrations were below the lowest value of healthy volunteers. Then we examined expressions of these miRNAs in cancer tissues compared with those in adjacent normal tissues from these eight patients. All the miRNAs obtained from formalin-fixed paraffin-embedded tissues were amplified, and found to be of good quality for amplification (data not shown). *MiR-106b* showed higher expression in primary GC tissues than normal mucosa in seven of the eight patients analysed (87.5%), whereas *let-7a* showed lower expression in seven patients (87.5%) (Table 1).

**Figure 3:**
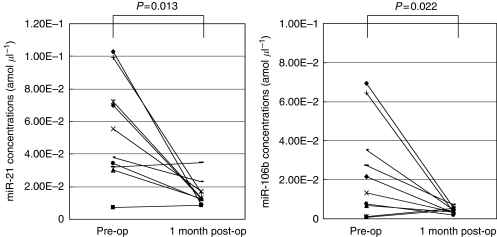
Comparison of plasma *miR-21* and *miR-106b* concentrations between pre- and post-operative samples from gastric cancer patients. Expressions of both miRNAs were significantly reduced in plasma samples obtained 1 month after surgical removal of the tumour.

Then, the concentrations of *miR-21* and *miR-106b* were analysed in paired pre- and post-operative plasma samples from 10 GC patients who underwent gastrectomy. Both miRNAs were significantly reduced in post-operative samples compared with the levels in pre-operative samples (*P*=0.013 and 0.022 respectively; Figure 3). These findings showed that primary cancer tissues and plasma samples from most patients showed similar tendencies concerning the miRNA levels, and indicated that the level of plasma miRNAs might reflect the expression level of tumour miRNAs.

### Large-scale validation on plasma samples

**Figure 4:**
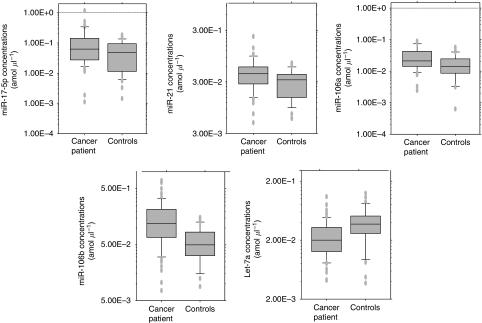
Box plots of the plasma miRNA concentrations in gastric cancer patients and controls. Plasma miRNA concentrations were significantly higher for *miR-17-5p* (*P*=0.05), *miR-21 (P*=0.006), *miR-106a* (*P*=0.008) and *miR-106b* (*P*<0.001) in the gastric cancer patients compared to those in controls, whereas *let-7a* was significantly lower in gastric cancer patients (*P*=0.002) . The upper and lower limits of the boxes and the lines inside the boxes indicate the 75th and 25th percentiles and the median respectively. The upper and lower horizontal bars denote the 90th and 10th percentiles respectively.

**Figure 5:**
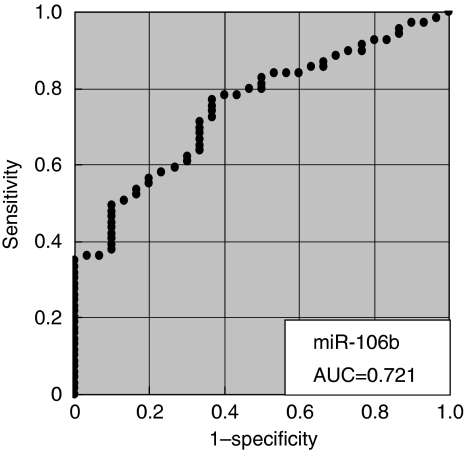
Receiver-operating characteristic (ROC) curve analysis in the concentration of *miR-106b* assay for detecting gastric cancer patients.

**Figure 6:**
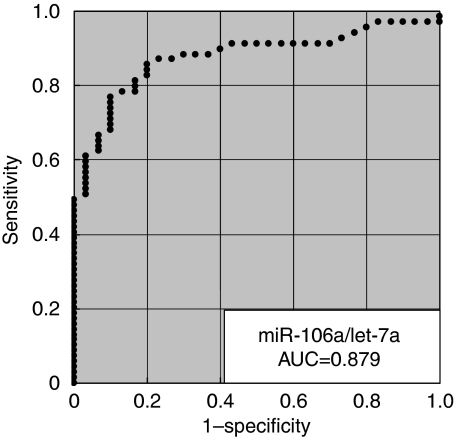
Receiver-operating characteristic (ROC) curve analysis of the ratio of *miR-106a/let-7a* assay for detecting gastric cancer patients.

In total, 69 GC patients were included in this study; 38 patients with TNM stage I, 13 with stage II, 14 with stage III and 4 with stage IV. We analysed another two miRNAs, *miR-17-5p* and *miR-106a*, for large-scale validation. The plasma concentrations of miRNAs, *miR-17-5p*, *miR-21*, *miR-106a* and *miR-106b*, were significantly higher in GC patients than in controls (*P*=0.006, 0.05, 0.008 and <0.001 respectively), whereas the concentration of *let-7a* was significantly lower in GC patients than in controls (*P*=0.002; Figure 4). Analyses of the ROC curves for plasma miRNAs showed that the AUC was greatest for *miR-106b* (AUC=0.721; Figure 5). To investigate more sensitive plasma diagnostic biomarkers, we analysed the ratio of circulating miRNA levels, dividing the plasma concentrations of *miR-17-5p*, *miR-21*, *miR-106a* and *miR-106b* by that of *let-7a*, and then, the ratio of *miR-106a*/*let-7a* showed the highest AUC of 0.879 (Figure 6). In this model, an optimal cut-off point was indicated at 0.536 with a sensitivity of 85.5% and a specificity of 80.0%. Other analyses of the ROC curves are shown in supplementary date (Supplementary Figures 1 and 2).

## Discussion

Numerous genetic and epigenetic alterations are known to be involved in tumourigenesis and tumour progression of various cancers. Several studies have identified tumour-specific alterations in plasma/serum nucleic acids of cancer patients, and have shown the potential of plasma circulating nucleic acids to be new non-invasive biomarkers in patients with various cancers (Sidransky, 1997; Anker *et al*, 2001; Taback and Hoon, 2004; Chan and Lo, 2007).

However, during the past decade, non-coding RNAs, so-called microRNAs (miRNAs), have also been shown to regulate gene expression by targeting mRNAs for translational repression or cleavage. Consequently, these miRNAs have recently been identified as new factors related to oncogenesis and tumour progression in various cancers (He *et al*, 2005, 2008; Lu *et al*, 2005; Calin and Croce, 2006).

Mitchell *et al* (2008) recently reported that miRNAs are detectable in plasma and that circulating miRNAs have the potential to be new biomarkers in patients with prostate cancers. They also showed the high stability of plasma miRNAs after prolonged incubation at room temperature and/or multiple freezing–thawing processes. In addition to this high stability, the characteristics of miRNAs such as tissue-specific miRNA signatures and the availability of many copies per cell would indicate potential advantages as biomarkers compared with those of other nucleic acids, such as circulating DNA and mRNA. In fact, accumulating reports also suggest the potential of miRNAs in the early detection of patients with several malignancies, such as lymphoma, colorectal cancer, tongue cancer and ovarian cancer (Lawrie *et al*, 2008; Wong *et al*, 2008; Ng *et al*, 2009; Resnick *et al*, 2009).

These findings prompted us to investigate the usefulness of miRNAs in patients with GCs. In this report, we selected four miRNAs (*miR-17-5p*, *miR-21*, *miR-106a* and *miR-106b*) that have been reported to be upregulated in GC, and also selected *let-7a*, which has been reported to be downregulated in GC (Zhang *et al*, 2007, 2008; Chan *et al*, 2008; Motoyama *et al*, 2008; Guo *et al*, 2009; Kim *et al*, 2009). First, we confirmed a linear correlation between the logarithm of the amount of input synthetic miRNA and the cycle threshold value on real-time PCR as well as the feasibility of extracting total RNA and amplifying specific miRNA in plasma samples. On the basis of these results, we used the absolute concentration method for measuring plasma miRNAs in this study. Some investigators have determined quantities of plasma miRNAs by comparing internal control miRNAs (Ng *et al*, 2009; Resnick *et al*, 2009). However, which miRNA is suitable as an internal control for plasma assay remains controversial.

Next, we investigated whether plasma miRNAs could be released from the primary gastric tumours. The comparison between expressions of miRNA in plasma and primary tumour tissues demonstrated that plasma and primary GC tissue samples showed similar tendencies concerning the expression of miRNAs in almost all cases. Each patient, however, showed a different pattern of miRNA levels, high plasma *miR-106b* with a low expression in GC tissue and low plasma *let-7a* with a high expression in cancer tissue. Although the reason for these discrepancies remains to be identified, one possible explanation for this finding is the heterogeneity of the primary tumours. We also measured circulating miRNAs in paired plasma before and 1 month after surgical removal of the tumours, to confirm tumour release of the circulating miRNAs. As a result, the concentrations of *miR-21* and *miR-106b* were significantly reduced postoperatively in patients with high pre-operative plasma *miR-21* and *miR-106b*. The kinetics and metabolism of the plasma miRNAs have not yet been clearly elucidated, however 1 month seems to be sufficient time for clearance of the circulating miRNAs.

Finally, we performed a large-scale study by increasing the number of plasma samples from GC patients and healthy volunteers to validate the diagnostic potential of plasma miRNAs. We found that the plasma concentrations of miRNAs, *miR-17-5p*, *miR-21*, *miR-106a* and *miR-106b* were significantly higher in GC patients than in controls, and the ROC analysis showed the greatest AUC value for *miR-106b*. Although, the concentration of *let-7a* was significantly lower in GC patients than in controls, contrary to our expectation, we have also found that a certain miRNA that was reported to be downregulated in primary cancer tissues showed a lower plasma expression level in oesophageal cancer patients (data not shown). Because circulating miRNAs are considered to have been released from cancer tissues as well as from normal tissues, the majority are expected to have originated from normal tissues. Several recent reports suggested that plasma miRNAs might be protected in a complex with other molecules, such as exosomes, proteins and lipids (Valadi *et al*, 2007; Mitchell *et al*, 2008). The protection of miRNAs might be greater in GC patients than controls. An alternative hypothesis is that a lower plasma expression of certain miRNAs might be due to alterations in miRNA expressions in normal tissues of cancer patients by unknown mechanisms.

Determining the expression ratios of genes or miRNAs has been reported to be a useful technique to improve diagnostic potential (Gordon *et al*, 2002; Avissar *et al*, 2009). Therefore, we also investigated the miRNA expression ratios by dividing the plasma concentration of upregulated miRNA (*miR-17-5p*, *miR-21*, *miR-106a* and *miR-106b*) by that of downregulated miRNA (*let-7a*) to improve the sensitivity and specificity of plasma miRNA assay for use as diagnostic biomarkers. Then, the ratio of *miR-106a*/*let-7a* showed the highest AUC value of 0.879 in this study, and which would be satisfactory for clinical application.

Plasma miRNA assays have several potential clinical uses: screening patients at high risk for GC and monitoring disease recurrence during the follow-up period after gastrectomy. These miRNA biomarkers might also be powerful and useful for confirming the completeness of tumour resection and evaluating the efficacy of adjuvant therapies if the elimination clearance of plasma miRNAs can be elucidated.

In conclusion, plasma miRNAs may be available as a new marker for GC. Further prospective clinical trials using a variety of plasma miRNAs should be carried out to define the usefulness of the assay for each potential application.

## Figures and Tables

**Table 1:** Expression of mature miRNAs in gastric cancer tissue *vs* those in normal tissue

	***miR-106b***	***let-7a***
Case 1	1.344	0.722
Case 2	1.357	0.681
Case 3	1.241	0.880
Case 4	0.346	0.515
Case 5	3.812	3.659
Case 6	9.966	0.129
Case 7	1.608	0.139
Case 8	2.897	0.260
Rate of higher expression level in gastric cancer tissue	87.5%	12.5%
Rate of lower expression level in gastric cancer tissue	12.5%	87.5%
