# Supplementary material for: Circulating microRNAs in plasma of patients with gastric cancers
Source: Br J Cancer. 2010 Mar 16;102(7):1174–9. doi: 10.1038/sj.bjc.6605608 (PMC2853097; doi:10.1038/sj.bjc.6605608)
Supplement: Supplementary Figures and Table [file 6605608x1.ppt]

## Slide 1
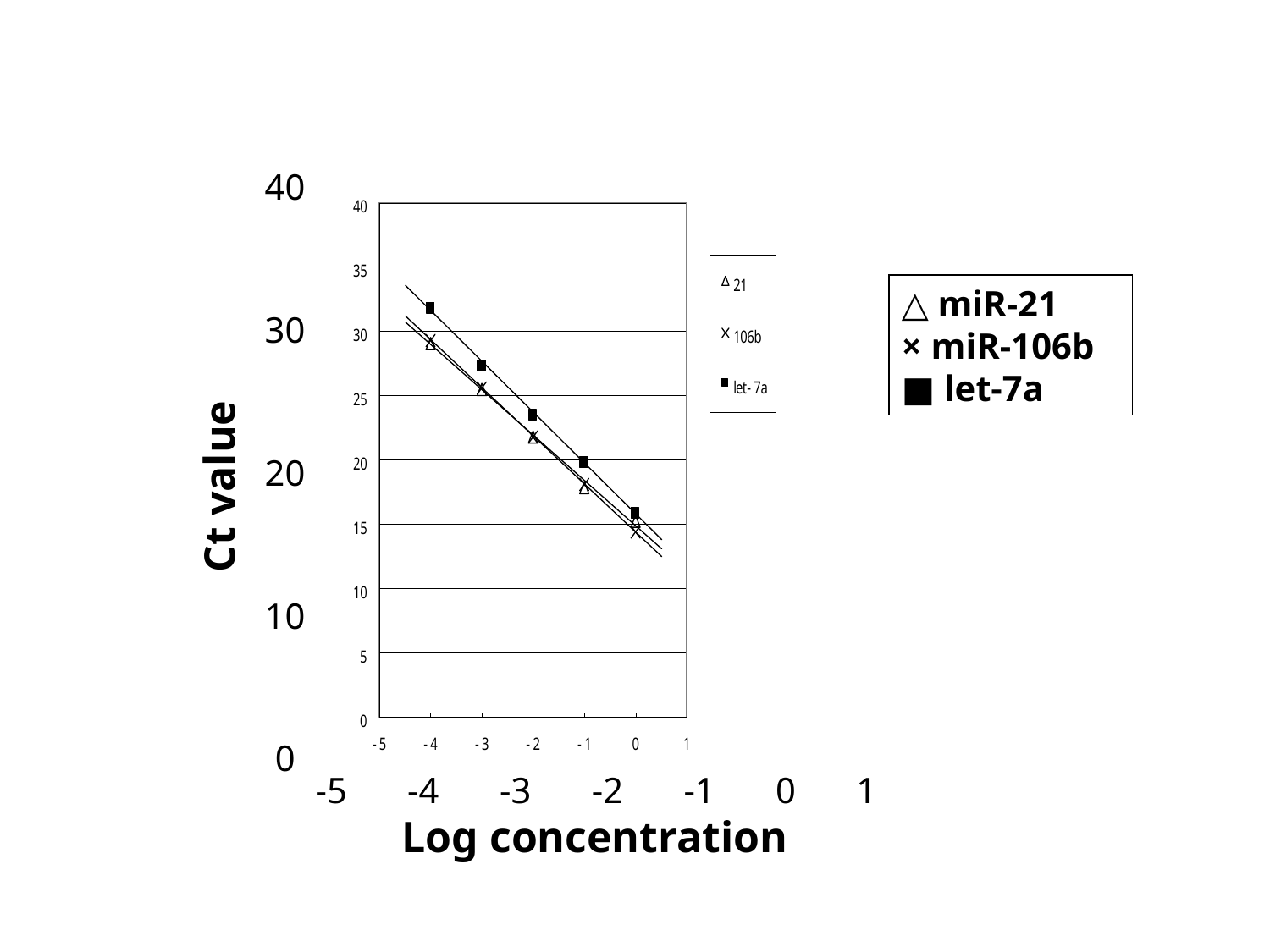

40
30
20
10
0
△ miR-21
× miR-106b
■ let-7a
Ct value
1
-5
-4
-3
-2
-1
0
Log concentration

## Slide 2
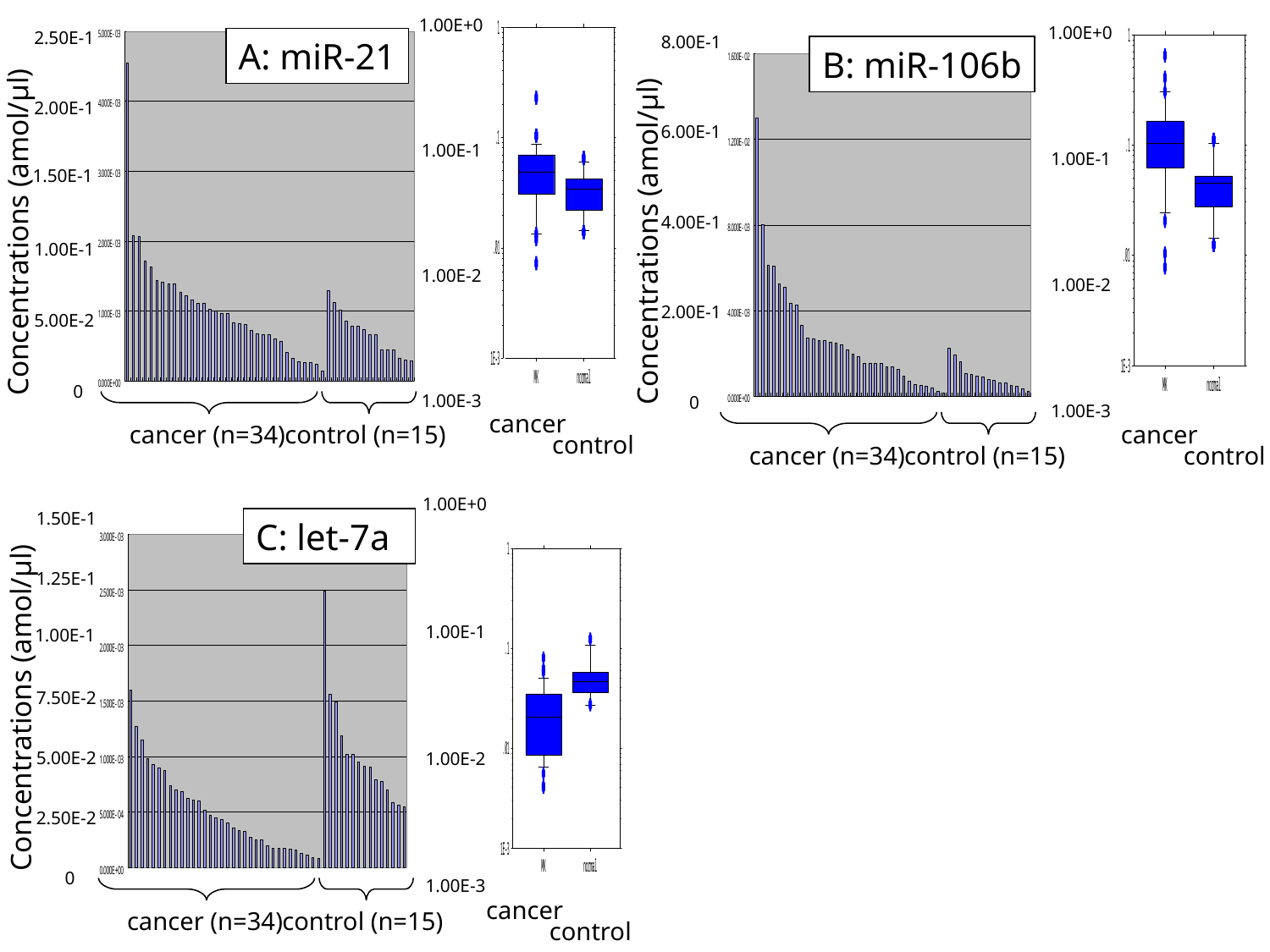

1.00E+0
1.00E-1
1.00E-2
1.00E-3
2.50E-1
2.00E-1
1.50E-1
1.00E-1
5.00E-2
0
Concentrations (amol/μl)
cancer (n=34)
control (n=15)
cancer
control
A: miR-21
1.00E+0
1.00E-1
1.00E-2
1.00E-3
8.00E-1
6.00E-1
4.00E-1
2.00E-1
0
Concentrations (amol/μl)
cancer (n=34)
control (n=15)
cancer
control
B: miR-106b
1.00E+0
1.00E-1
1.00E-2
1.00E-3
1.50E-1
1.25E-1
1.00E-1
7.50E-2
5.00E-2
2.50E-2
0
Concentrations (amol/μl)
cancer (n=34)
control (n=15)
cancer
control
C: let-7a

## Slide 3
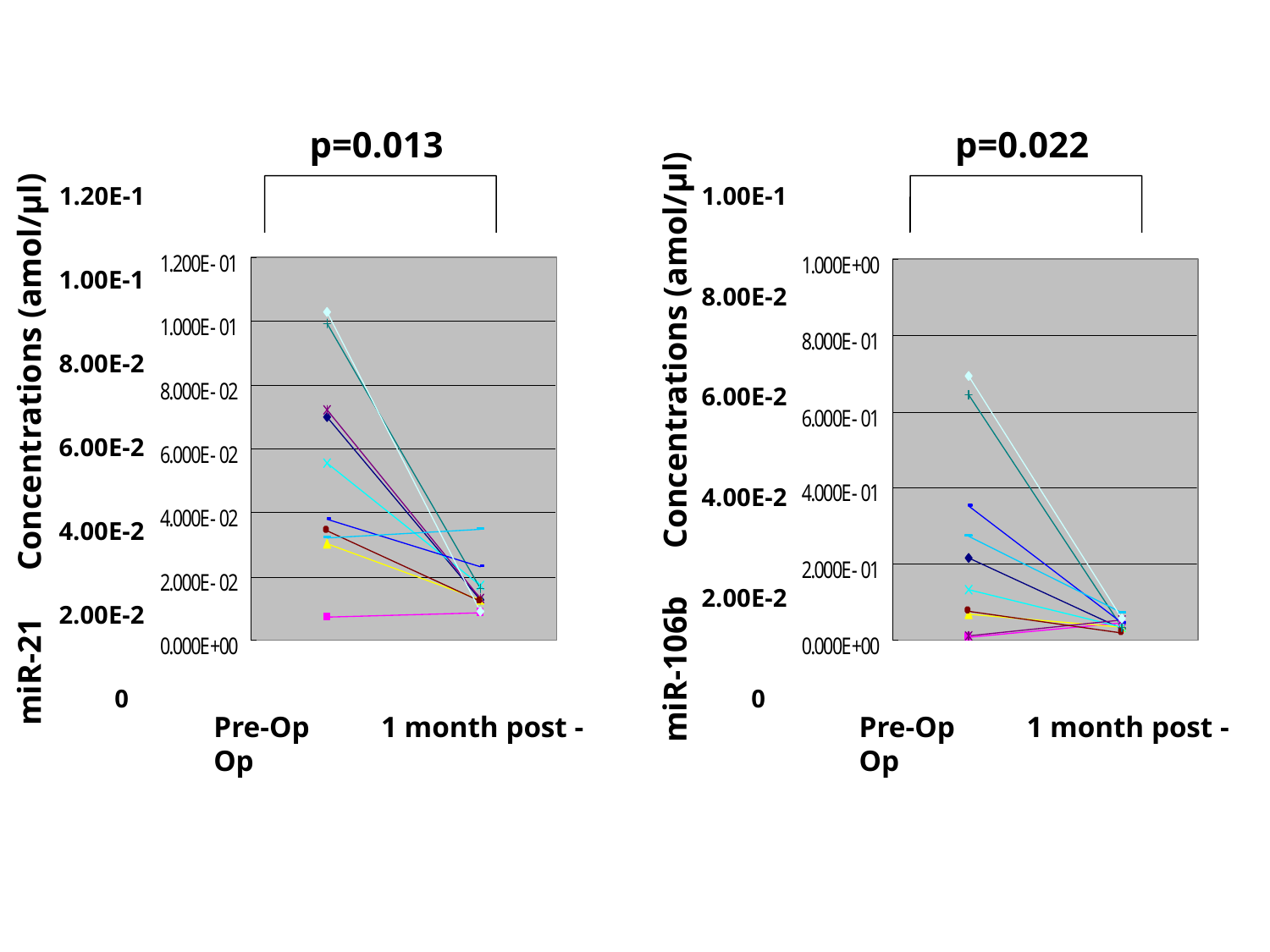

p=0.013
1.20E-1
1.00E-1
8.00E-2
6.00E-2
4.00E-2
2.00E-2
0
miR-21　Concentrations (amol/μl)
Pre-Op 　1 month post -Op
 p=0.022
1.00E-1
8.00E-2
6.00E-2
4.00E-2
2.00E-2
0
miR-106b　Concentrations (amol/μl)
Pre-Op 　1 month post -Op

## Slide 4
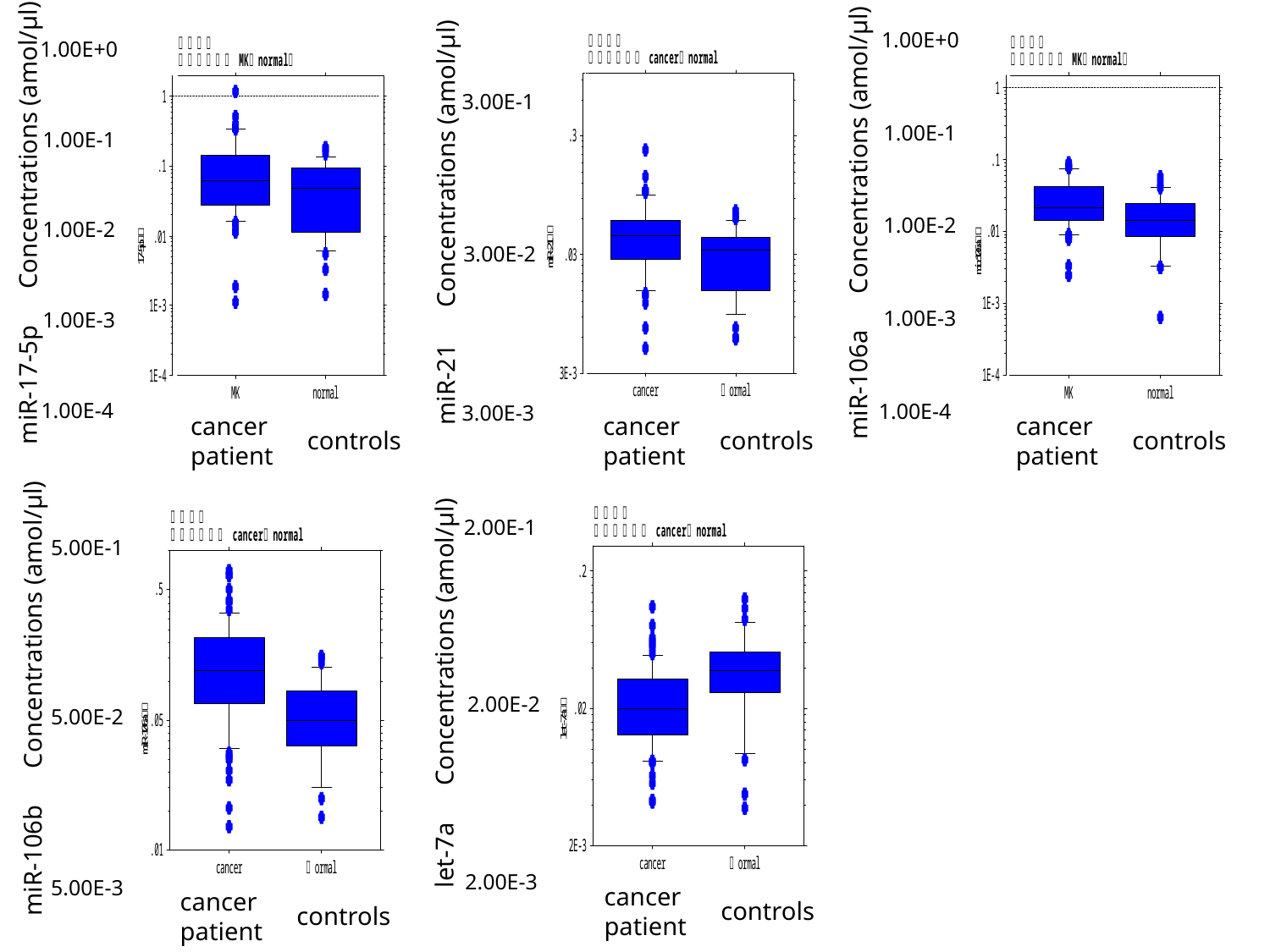

1.00E+0
1.00E-1
1.00E-2
1.00E-3
1.00E-4
miR-17-5p　Concentrations (amol/μl)
cancer
patient
controls
1.00E+0
1.00E-1
1.00E-2
1.00E-3
1.00E-4
miR-106a　Concentrations (amol/μl)
cancer
patient
controls
3.00E-1
miR-21　Concentrations (amol/μl)
3.00E-2
3.00E-3
cancer
patient
controls
5.00E-1
5.00E-2
5.00E-3
miR-106b　Concentrations (amol/μl)
cancer
patient
controls
2.00E-1
2.00E-2
2.00E-3
let-7a　Concentrations (amol/μl)
cancer
patient
controls

## Slide 5
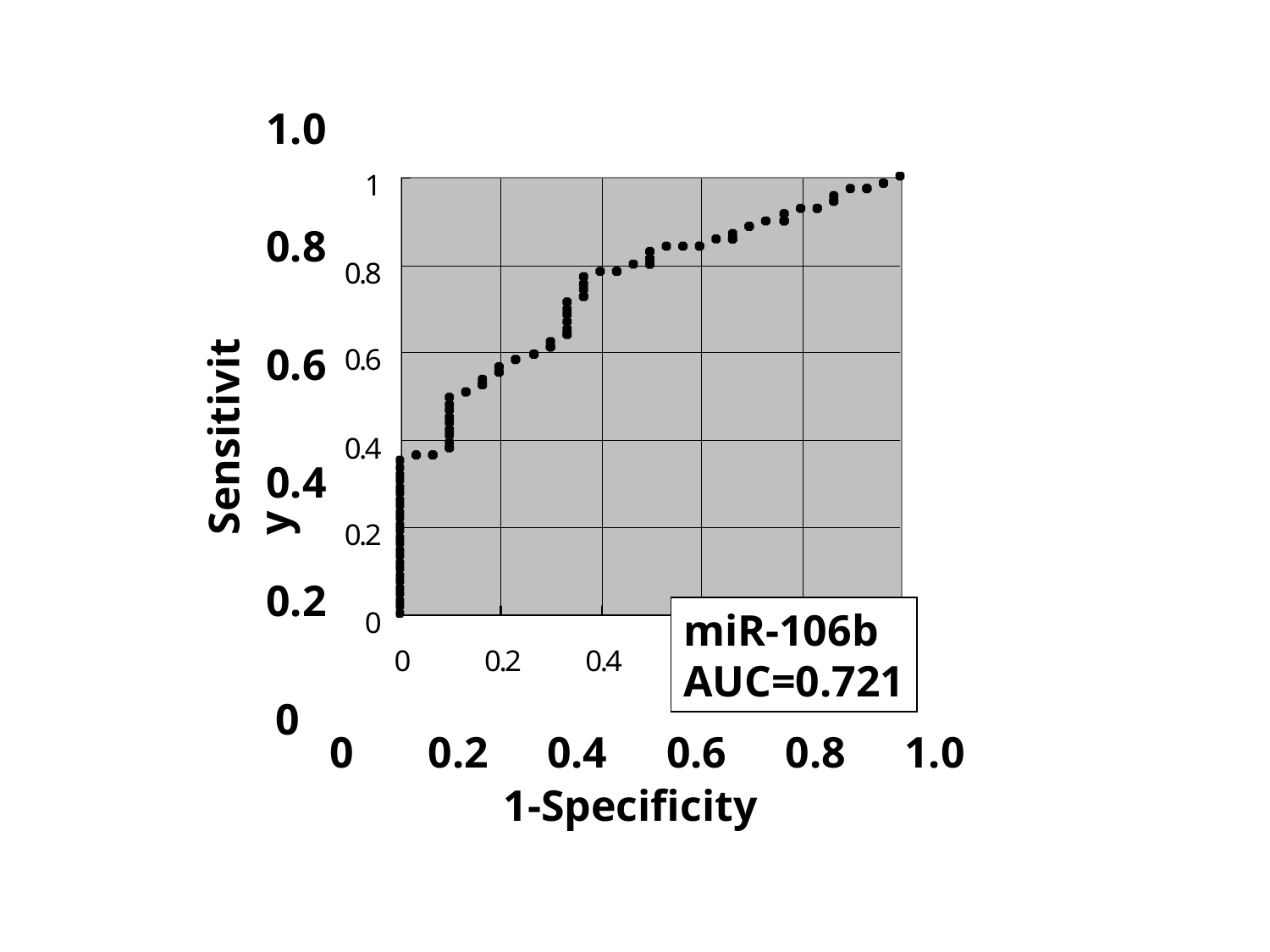

1.0
0.8
0.6
0.4
0.2
0
Sensitivity
miR-106b
AUC=0.721
0
0.2
0.4
0.6
0.8
1.0
1-Specificity

## Slide 6
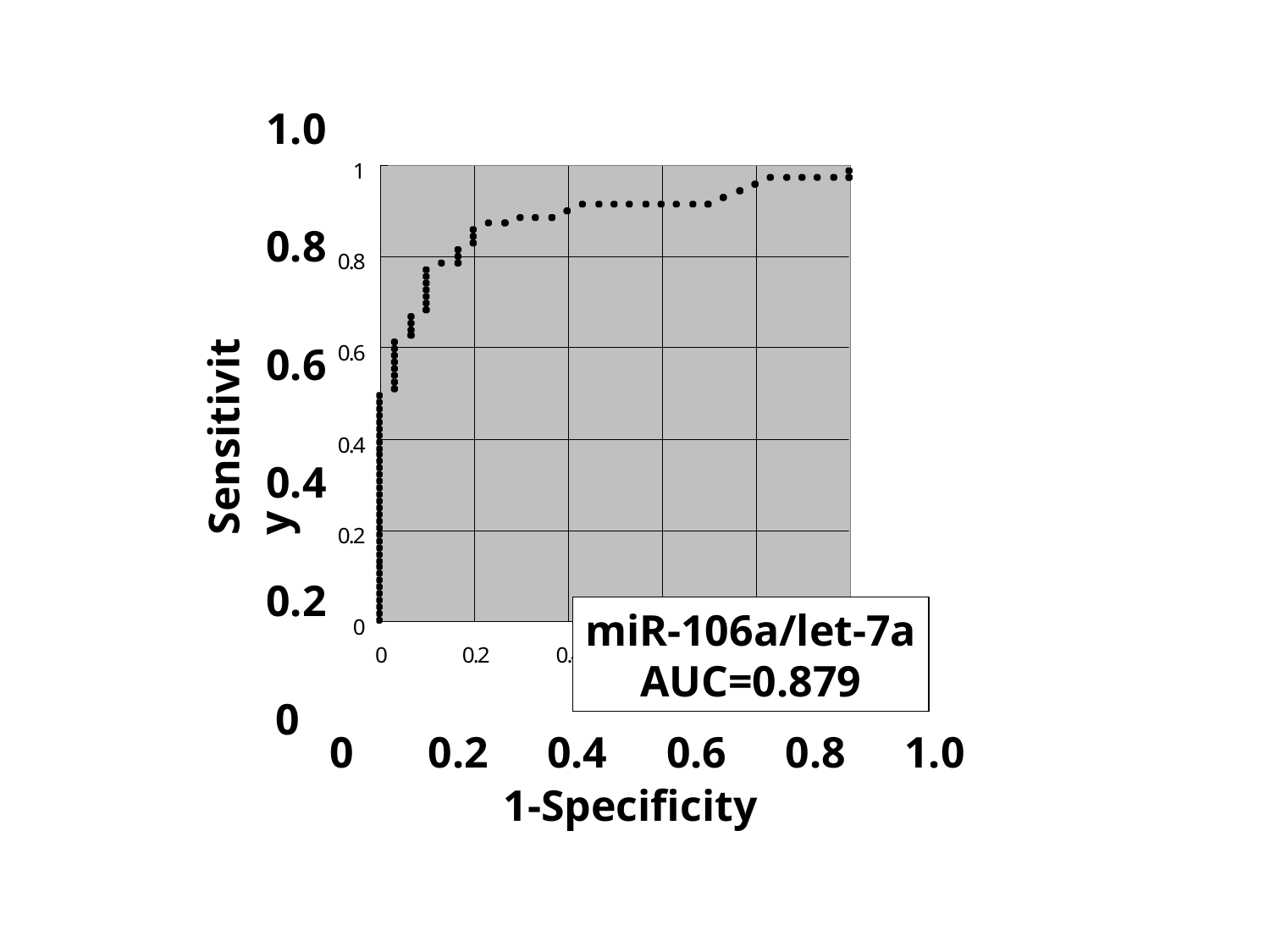

1.0
0.8
0.6
0.4
0.2
0
Sensitivity
miR-106a/let-7a
AUC=0.879
0
0.2
0.4
0.6
0.8
1.0
1-Specificity

## Slide 7
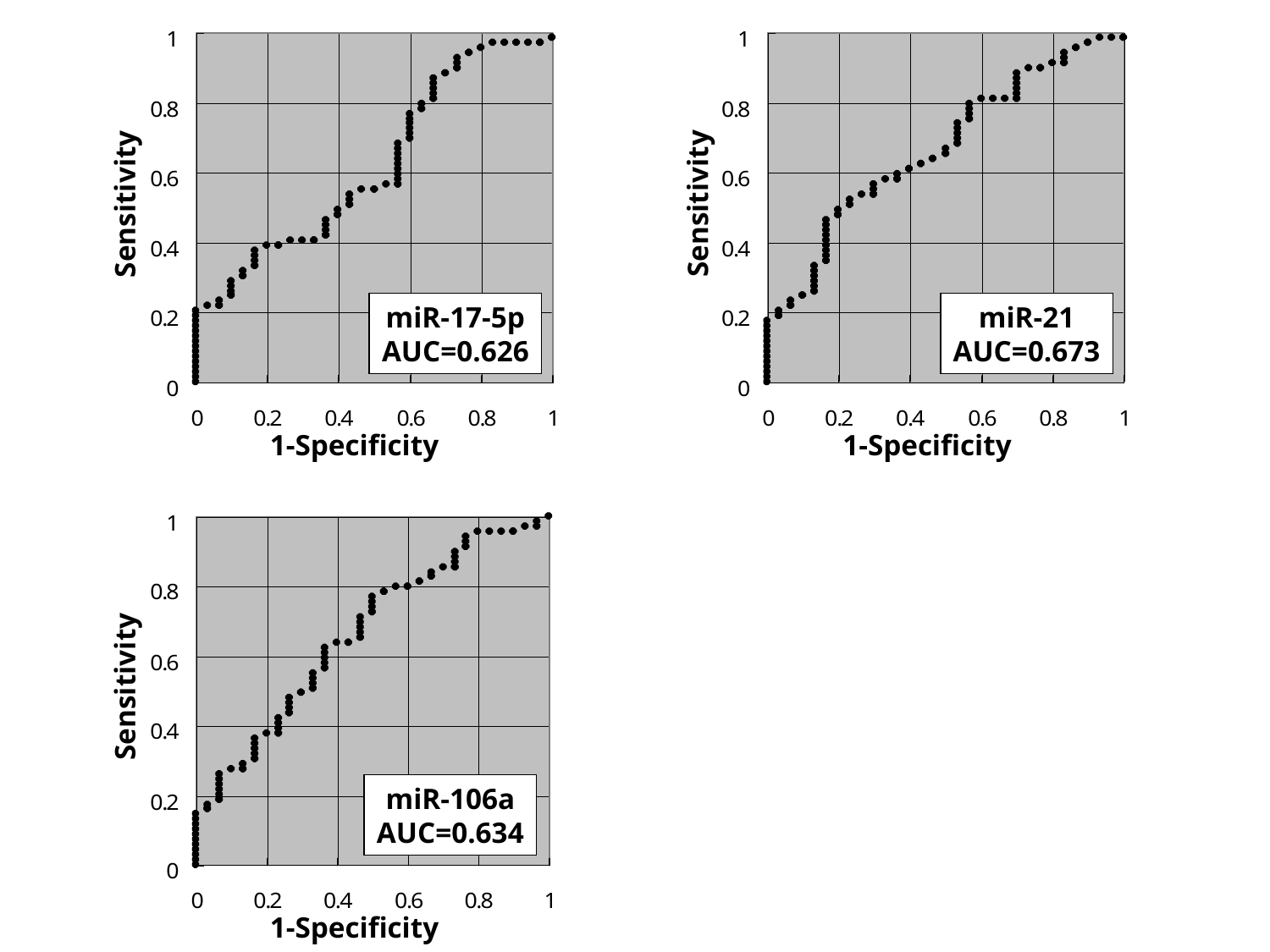

Sensitivity
miR-17-5p
AUC=0.626
1-Specificity
Sensitivity
miR-21
AUC=0.673
1-Specificity
Sensitivity
miR-106a
AUC=0.634
1-Specificity

## Slide 8
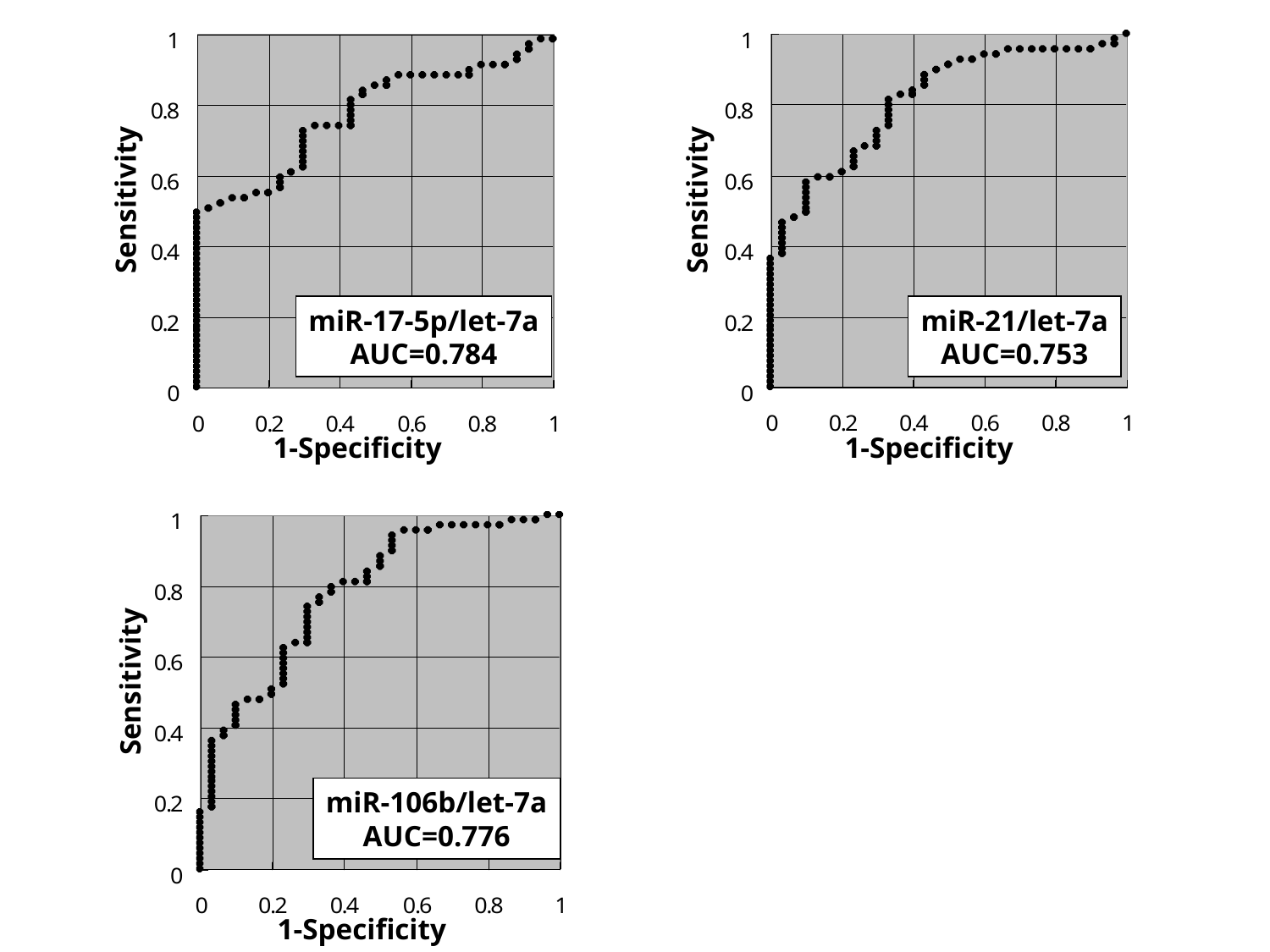

Sensitivity
miR-21/let-7a
AUC=0.753
1-Specificity
Sensitivity
miR-17-5p/let-7a
AUC=0.784
1-Specificity
Sensitivity
miR-106b/let-7a
AUC=0.776
1-Specificity

## Slide 9
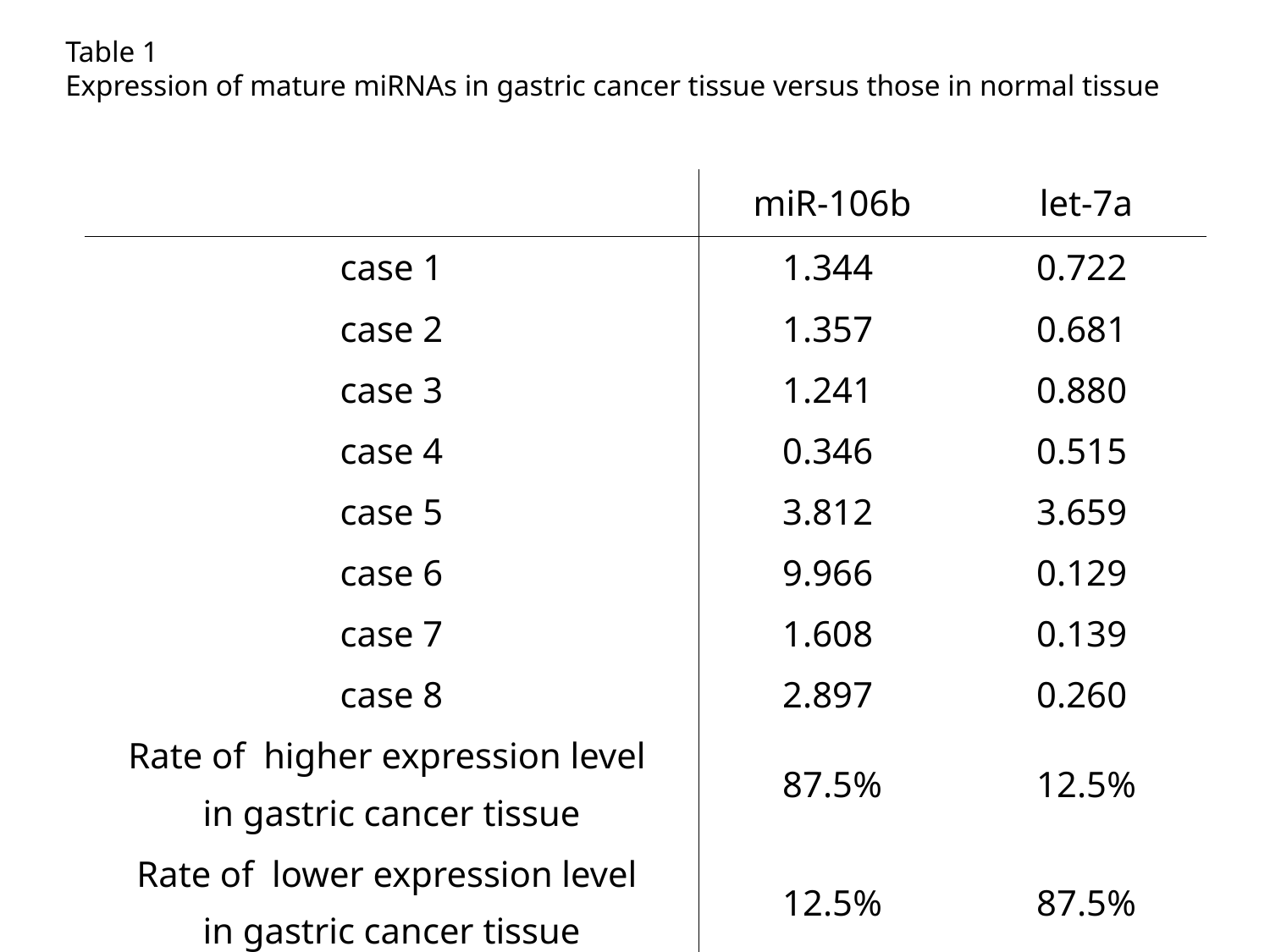

Table 1
Expression of mature miRNAs in gastric cancer tissue versus those in normal tissue
| | miR-106b | let-7a |
| --- | --- | --- |
| case 1 | 1.344 | 0.722 |
| case 2 | 1.357 | 0.681 |
| case 3 | 1.241 | 0.880 |
| case 4 | 0.346 | 0.515 |
| case 5 | 3.812 | 3.659 |
| case 6 | 9.966 | 0.129 |
| case 7 | 1.608 | 0.139 |
| case 8 | 2.897 | 0.260 |
| Rate of higher expression level in gastric cancer tissue | 87.5% | 12.5% |
| Rate of lower expression level in gastric cancer tissue | 12.5% | 87.5% |
