# Supplementary material for: Circulating microRNAs in plasma of patients with gastric cancers
Source: Br J Cancer. 2010 Mar 16;102(7):1174–9. doi: 10.1038/sj.bjc.6605608 (PMC2853097; doi:10.1038/sj.bjc.6605608)
Supplement: Supplementary Figure Legends [file 6605608x2.doc]

**Figure Legend**

**Figure 1**

**Standard curve of *miR-21*, *miR-106b*, *let-7a* using synthetic microRNAs**

Ten-fold serial dilution of synthetic microRNA was used to generate the standard curves. Linearity was confirmed within these concentrations, ranging from 1fmol to 0.0001 fmol. (*miR-21*: y = -3.7544x + 14.318 (R2 = 0.999), *miR-106b*: y = -3.9849x + 15.645 (R2 = 0.998), *let-7a*: y = -3.4988x + 14.875 (R2 = 0.999))

**Figure 2**

**Plasma miRNAs concentration in the initial analysis**

Real-time RT-PCR assay, circulating plasma miRNAs (*miR-21*, *miR-106b*, and *let-7a*) were detectable and amplified in all samples from 34 gastric cancer patients and 15 healthy volunteers. The concentrations of *miR-106b* and *let-7a* were significantly higher and lower in plasma from gastric cancer patients than in that from healthy controls (p=0.002, p<0.001, respectively). However, there was no significant difference in the concentration of *miR-21* between gastric cancers patients and controls although it tended to be higher in gastric cancer patients (p=0.088). The upper and lower limits of the boxes and the lines inside the boxes indicate the 75th and 25th percentiles and the median, respectively. The upper and lower horizontal bars denote the 90th and 10th percentiles, respectively.

**Figure 3**

**Comparison of plasma *miR-21* and *miR-106b* concentrations between pre- and post-operative samples from gastric cancer patients**

Expressions of both miRNAs were significantly reduced in plasma samples obtained 1-month after surgical removal of the tumor.

**Figure 4**

**Box plots of the plasma miRNA concentrations in gastric cancer patients and controls**

Plasma miRNA concentrations were significantly higher for *miR-17-5p*(p=0.05), *miR-21(*p=0.006), *miR-106a*(p=0.008) and *miR-106b*(p<0.001) in the gastric cancer patients compared to those in controls, while *let-7a* was significantly lower in gastric cancer patients(p=0.002) . The upper and lower limits of the boxes and the lines inside the boxes indicate the 75th and 25th percentiles and the median, respectively. The upper and lower horizontal bars denote the 90th and 10th percentiles, respectively.

**Figure 5**

**Receiver-operating characteristic (ROC) curve analysis in the concentration of *miR-106b* assay for detecting gastric cancer patients**

**Figure 6**

**Receiver-operating characteristic (ROC) curve analysis of the ratio of *miR-106a/let-7a* assay for detecting gastric cancer patients**

**Supplementary Figure 1**

**Receiver-operating characteristic (ROC) curve analysis in the concentration of *miR-17-5p*, *miR-21*, *and miR-106a* assays for detecting gastric cancer patients**

**Supplementary Figure 2**

**Receiver-operating characteristic (ROC) curve analysis of the ratio of *miR-17-5p/let-7a*, *miR-21/let-7a*, and *miR-106b/let-7a* assays for detecting gastric cancer patients**
